# Effectiveness of Six Irrigation Techniques With Sodium Hypochlorite in Tissue Dissolution

**DOI:** 10.7759/cureus.39208

**Published:** 2023-05-18

**Authors:** He Liu, Ya Shen, Markus Haapasalo

**Affiliations:** 1 Division of Endodontics, Department of Oral Biological and Medical Sciences, Faculty of Dentistry, University of British Columbia, Vancouver, CAN

**Keywords:** irrigation, er:yag laser, er:cr:ysgg laser, tissue dissolution, gentlewave

## Abstract

Introduction: Complete removal of pulp tissue by mechanical instrumentation remains a challenge. The study aimed to assess the efficacy of six irrigation techniques using 3% sodium hypochlorite (NaOCl) in dissolving tissue.

Materials and methods: Seventy standardized fragments of bovine muscle tissue were divided into seven groups and exposed to different irrigation devices, with a maximum of 10 minutes of irrigation at room temperature. The devices included erbium-doped yttrium-aluminum-garnet (Er:YAG) laser (LightWalker® ST-E; Fotona d.o.o., Ljubljana, Slovenia), erbium, chromium-doped yttrium, scandium, gallium and garnet (Er,Cr:YSGG) laser A (EdgePRO™; Biolase Technology Inc., San Clemente, California, United States), Er,Cr:YSGG laser B (WaterLase iPlus®; Biolase, California, United States), multisonic ultracleaning system (MUS) GentleWave® system with CleanFlow™ handpiece (Sonendo, Inc., Laguna Hills, California, United States), passive ultrasonic irrigation (PUI), EndoUltra ultrasonic activator (Vista Apex Dental Products, Racine, Wisconsin, United States), and conventional needle irrigation (CNI), a 29G NaviTip irrigation needle at a flow rate of 5 mL/min (Ultradent Products, Inc., Utah, United States), with 3% NaOCl without agitation as the control. For samples that dissolved completely within 10 minutes, the dissolution time was recorded, and for other samples, the tissue weight was measured after the 10-minute irrigation experiment. The tissue dissolution rate (%/s) was calculated for all experiments.

Results: MUS had the fastest tissue dissolution rate (*P* < 0.01), at 2.986% ± 0.395% per second. The laser systems had dissolution rates of 0.388% ± 0.062% per second for Er:YAG laser, 0.316% ± 0.042% for Er,Cr:YSGG laser A, and 0.258% ± 0.018% for Er,Cr:YSGG laser B. CNI and PUI showed the slowest tissue dissolution rates.

Conclusions: MUS with CleanFlow, followed by Er:YAG and Er,Cr:YSGG lasers, achieved a significantly faster tissue dissolution rate than conventional irrigation methods of PUI and CNI tested. MUS with CleanFlow can greatly improve the effectiveness of NaOCl in tissue dissolution.

## Introduction

Complete removal of pulp tissue in endodontic treatment remains challenging due to the complex anatomies and irregular morphology of the root canal system [1,2]. Root canal irrigation is crucial for removing tissue remnants left by mechanical debridement [3,4]. Sodium hypochlorite (NaOCl) is considered the first choice as an irrigant solution in endodontic treatment because it is the only solution used in endodontics that can dissolve organic matter, such as necrotic pulp tissue and root canal biofilm, both of which are crucial for the success of the treatment [5,6]. It also has excellent antimicrobial activity. Furthermore, NaOCl is a relatively low-cost and readily available solution. It is easy to use, and the irrigant can be delivered through a variety of irrigation devices, such as syringes, needles, and ultrasonic instruments. Previous studies have indicated that laser-activated irrigation techniques, such as erbium-doped yttrium aluminum garnet (Er:YAG) and erbium, chromium, yttrium, scandium, gallium garnet (Er,Cr:YSGG) lasers, can enhance the effectiveness of NaOCl in tissue dissolution [7,8]. A new Er,Cr:YSGG laser system, EdgePRO™ (Biolase Technology Inc., San Clemente, California, United States), has been introduced recently for cleaning, debridement, and disinfection of the root canal system. However, there is no published study on the effectiveness of EdgePRO with NaOCl in tissue dissolution.

A multisonic ultracleaning system (MUS), GentleWave® (Sonendo Inc, Laguna Hills, California, United States) has been developed for cleaning the root canal system. It generates a broad spectrum of soundwaves and, according to some studies, can create advanced fluid dynamics [9-11]. A previous study has shown that MUS using a molar handpiece with NaOCl dissolves soft tissue much faster than conventional irrigation methods using syringe-needle irrigation or ultrasound [9]. Recently, a new handpiece called CleanFlow™ ((Sonendo, Inc.) was introduced in MUS. Unlike previous handpiece designs, no part of the CleanFlow handpiece is placed in the access cavity or pulp chamber, and the mechanism by which fluid dynamics is created is also different. However, there is no data on the soft tissue-dissolving capacity of MUS with the CleanFlow handpiece. Moreover, there are no in vitro or in vivo studies that have compared lasers and MUS.

Therefore, the aim of this study was to quantitatively evaluate and compare the in vitro soft tissue-dissolving effectiveness of Er:YAG laser, Er,Cr:YSGG laser, MUS with the CleanFlow handpiece, and two conventional root canal irrigation methods. The hypothesis tested was that there was significant difference in the effectiveness of irrigation activation techniques tested with 3% NaOCl in tissue dissolution.

## Materials and methods

Devices used for irrigation

The endodontic devices and instruments used in the experiment included the following: (i) LightWalker® ST-E laser system (Fotona d.o.o., Ljubljana, Slovenia), (ii) EdgePRO laser system, (iii) WaterLase iPlus® laser system (Biolase Technology Inc.), (iv) MUS GentleWave® system with CleanFlow™ handpiece, (v) passive ultrasonic activation (PUI) with a cordless ultrasonic activator (EndoUltra; Vista Apex Dental Products, Racine, Wisconsin, United States) with a NiTi activation tip (size 20, 0.02 taper), and (vi) conventional needle irrigation (CNI) with a 29G open-ended syringe needle (NaviTip; Ultradent Products, Inc., South Jordan, Utah, United States). The control group consisted of 3% NaOCl without any agitation.

Tissue specimen preparation

Seventy pieces of bovine muscle tissue were used in this study. The same fresh bovine meat was used to prepare the tissue specimens, which were then stored frozen at -20°C and left to thaw at room temperature before being cut into equal-sized pieces measuring 2 x 2 x 1 mm using a stainless-steel blade (Cincinnati Surgical Company Inc., Cincinnati, Ohio, United States). After blotting dry with a paper towel, the tissue specimen mass was measured using a calibrated electronic balance (FX-300; A&D Company Ltd, Toshima City, Tokyo, Japan) to ensure that each tissue sample had an initial mass of 10 ± 0.5 mg. The tissue samples were then divided into seven groups (n=10 each): Er:YAG laser (LightWalker ST-E), Er,Cr:YSGG laser A (EdgePRO), Er,Cr:YSGG laser B (WaterLase iPlus), MUS, PUI (EndoUltra) with a NiTi activation tip (size 20, 0.02 taper), conventional needle irrigation (CNI) using a positive-pressure 29G NaviTip irrigation needle at a flow rate of 5 mL/min, and a control group (no agitation). There were no significant differences in the tissue sample mass used for each group (*P* > 0.05).

Tissue dissolution

To deliver the irrigant to the experimental apparatus, a 60 mL syringe filled with 3% NaOCl was connected to a 29G NaviTip irrigation needle using a Luer lock connection to Tygon ST tubing (Ismatec, Wertheim, Germany). A digitally controlled syringe pump (Fusion 200; Chemyx Inc., Stafford, Texas, United States) was used to ensure a precise flow rate of 5 mL/min. Before each use, the syringe pump and needle flow rate were calibrated as previously described [9]. To prevent the tissue specimen in the lower portion of the glass tube from being flushed out during the agitation process, a thin nylon mesh was inserted inside the tube, 10 mm from the top end of the glass tube, to mimic a pulp chamber and provide space for the different technologies to work. A thin glass tube was set below the nylon mesh to help stabilize the tissue specimen in the center.

**Figure 1:**
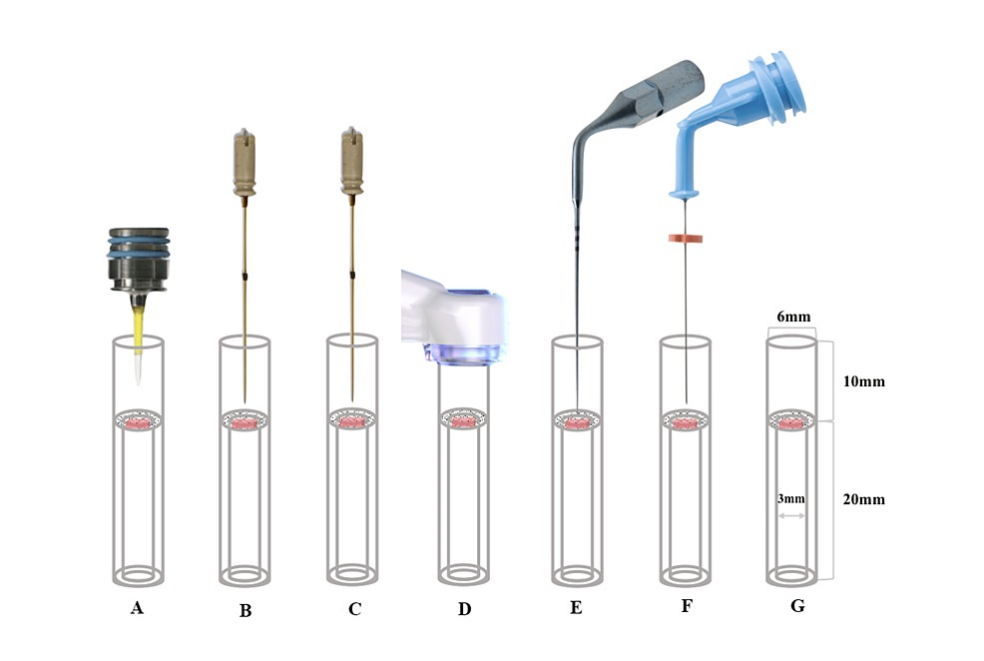
Schematic diagram drawing showing irrigation techniques tested in tissue dissolution. A: Er:YAG laser; B: Er,Cr:YSGG laser A; C: Er,Cr:YSGG laser B; D: MUS; E: PUI; F: CNI; G: Control MUS: multisonic ultracleaning system; PUI: passive ultrasonic irrigation; CNI: conventional needle irrigation; Er:YAG: erbium-doped yttrium-aluminum-garnet; Er,Cr:YSGG: erbium, chromium-doped yttrium, scandium, gallium, and garnet

Figure 1 illustrates the experimental setup for measuring tissue dissolution. The volume of the upper and lower tubes was 0.28 mL and 0.14 mL, respectively. Furthermore, to ensure that the same amount of suction was provided for all devices, suction was provided by the MUS at the top of the model.

In the three laser groups, PUI, CNI, and control groups, the 29G NaviTip irrigation needle was placed into the upper portion of the glass vial at a depth of 3 mm along the wall and continuously delivered 3% NaOCl solution into the upper portion at 5 mL/min, as described above. In the MUS group, the 29G NaviTip irrigation needle was not used. If the tissue sample was completely dissolved visually within 10 minutes, the time (in seconds) it took to dissolve the tissue was recorded. For all other tissue samples that were not completely dissolved, the samples were blotted dry with a paper towel, and the remaining tissue mass was measured and recorded.

Er:YAG Laser

The LightWalker ST-E console was set to SSP mode at 15 Hz, 20 mJ, and 0.3W, and the air-water spray was turned off. An experimental photon-induced photoacoustic streaming (PIPS) tip, which was 9 mm long and 600 μm in diameter, was used. The tip was positioned centrally in the upper portion of the test tube, 5 mm away from the tissue sample, and continuously activated.

Er,Cr:YSGG Laser A

The EdgePRO console was set to 50 Hz, 25 mJ, and 1.25 W, with the air-water spray turned off. A ONE-25 tip was centrally placed into the upper portion of the test tube, 3 mm away from the tissue sample, and activated continuously.

Er,Cr:YSGG Laser B

The WaterLase iPlus console was set to 50 Hz, 25 mJ, and 1.25 W, and the air-water spray was turned off. A RFT2-25 tip was positioned at the center of the upper part of the test tube, 3 mm away from the tissue sample, and activated continuously.

MUS

In the experiment, a CleanFlow handpiece from the GentleWave system was used. To maintain appropriate fluid dynamics, the space between the handpiece and the upper end of the glass tube was sealed with Loctite 4311 (Henkel AG & Co., Düsseldorf, Germany) to ensure an airtight seal. The CleanFlow handpiece was connected to the GentleWave console following the manufacturer's instructions. The vacuum created was due to the mechanism of action generated by the GentleWave console, which was set and operated according to the manufacturer's recommendations. Approximately 45 mL of 3% NaOCl was delivered into the access cavity per minute.

PUI

A #20/0.2 titanium activator tip was centrally positioned in the upper part of the test tube, at a distance of 1 mm from the tissue sample and activated continuously at a frequency of 40,000 Hz.

CNI 

A 29G NaviTip irrigation needle, which generated a positive pressure, was positioned at the center of the upper portion of the test tube and constantly delivered 3% NaOCl solution into the upper reservoir at a flow rate of 5 mL/min.

*Control Group* The test tube was filled with 3% NaOCl solution.

Data analysis

The tissue dissolution rate was calculated as the percent tissue mass loss per second. The data were analyzed using GraphPad Prism software version 9.4.1 (Graphpad Software, San Diego, California, United States). The normality of distribution and the homogeneity of variance were determined. The data of each group were normally distributed. One-way analysis of variance followed by Tukey post-hoc pairwise comparisons for multiple comparisons were used for the intergroup comparisons of the mean tissue dissolution rates of the different irrigation techniques. For all analyses, statistical significance was set at a predetermined level of α = 0.05.

## Results

The three laser systems and the MUS were able to completely dissolve the tissue specimens in less than 10 minutes, while none of the other irrigation protocols (PUI, CNI, and control group) were able to achieve complete dissolution within 10 minutes. The dissolution times for the different endodontic devices are presented in Table 1.

**Table 1:** Experimental parameters and tissue dissolution time (seconds) by 3% NaOCl using Er:YAG laser, Er,Cr:YSGG laser A, Er,Cr:YSGG laser B, MUS, PUI, CNI, and control groups (Mean ± SD; n = 10 for each group) MUS: multisonic ultracleaning system; N/A: not applicable; PUI: passive ultrasonic irrigation; CNI: conventional needle irrigation; PIPS: photon-induced photoacoustic streaming; Er:YAG: erbium-doped yttrium-aluminum-garnet; Er,Cr:YSGG: erbium, chromium-doped yttrium, scandium, gallium, and garnet

Group	Time (s)	Settings	Tip/handpiece	Distance between the tip and tissue specimen (mm)
Er:YAG	263.20 ± 40.62	SSP mode, 20mJ, 15Hz and 0.3W	PIPS tip	5
Er,Cr:YSGG A	320.60 ± 37.36	25mJ, 50Hz and 1.25W	ONE-25	3
Er,Cr:YSGG B	389.50 ± 26.73	25mJ, 50Hz and 1.25W	RFT2-25	3
MUS	34.00 ± 4.58	N/A	CleanFlow	7
PUI	>600	N/A	#20/0.2	1
CNI	>600	5mL/min	29G	3
Control	>600	N/A	N/A	N/A

**Figure 2:**
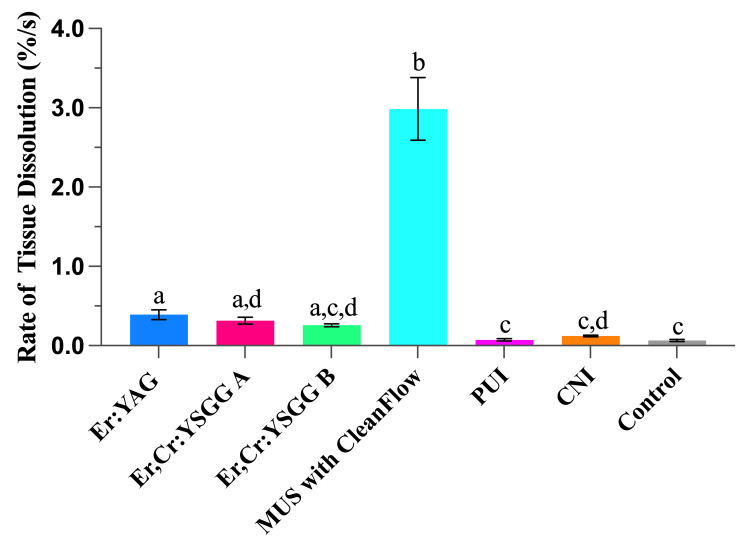
The rate of tissue dissolution (%/s) using Er:YAG laser, Er,Cr:YSGG laser A, Er,Cr:YSGG laser B, MUS, PUI, CNI, and control groups. Different letters indicate statistically significant differences between groups. MUS: multisonic ultracleaning system; PUI: passive ultrasonic irrigation; CNI: conventional needle irrigation; Er:YAG: erbium-doped yttrium-aluminum-garnet; Er,Cr:YSGG: erbium, chromium-doped yttrium, scandium, gallium, and garnet

Figure 2 shows the rates of tissue dissolution in 3% NaOCl for the devices tested at room temperature. The MUS had the fastest rate of tissue dissolution and dissolved the tissue specimen at a rate of 2.986% ± 0.395% per second. The MUS dissolution rate was significantly faster (*P* < 0.01) than any other device tested. The three laser systems had dissolution rates of 0.388% ± 0.062% per second for the Er:YAG laser, 0.316% ± 0.042%/s for the Er,Cr:YSGG laser A, and 0.258% ± 0.018%/s for the Er,Cr:YSGG laser B. The rate of tissue dissolution for MUS with 3% NaOCl was 7-11 times higher than the corresponding rates of the three laser systems tested in this study. Among the three laser systems, the Er:YAG laser achieved the fastest rate of tissue dissolution, followed by Er,Cr:YSGG laser A and Er,Cr:YSGG laser B (*P* < 0.01). CNI and PUI showed the slowest tissue dissolution rates, at 0.122% ± 0.010%/s and 0.073% ± 0.014%/s, respectively. The tissue dissolution rate in the control group (no agitation) was 0.065% ± 0.013%/s.

## Discussion

In this study, the MUS was found to be significantly more effective at tissue dissolution using 3% NaOCl compared to all other irrigation methods evaluated (*P* < 0.01). Therefore, the hypothesis that there was a significant difference in the effectiveness of irrigation activation techniques tested in tissue dissolution was accepted. The rate of tissue dissolution for the MUS with 3% NaOCl was 7-11 times higher than that of the three laser systems tested in this study. Conventional irrigation methods such as PUI, CNI, and no agitation were found to be the least effective. These results are consistent with a previous study in which the tissue dissolution by the MUS was found to be 8-15 times greater than that of conventional irrigation methods of PUI and CNI [9]. Another study also demonstrated the greater soft tissue cleaning capacity of MUS with minimal instrumentation compared to conventional rotary instrumentation and needle irrigation [10]. The larger difference between the MUS and conventional irrigation methods in the present study may be attributed to differences in the experimental model. However, the previous study did not include any lasers [9]. The high flow rate (approximately 45 mL/min) of hypochlorite used with the MUS may be a contributing factor to the faster tissue dissolution observed. While lasers do not have a built-in hypochlorite delivery system, dentists typically use a sequence of laser and syringe-needle irrigation, resulting in a volume of fresh NaOCl entering the tooth between 1 mL and 5 mL per minute, particularly when narrow needles of 29-31G are used. The acoustic energy and cavitation effect of the MUS and lasers may also play a role. Future studies should aim to measure the amount of energy released at the apical root canal.

Several previous studies have compared different characteristics of Er:YAG and Er,Cr:YSGG lasers [12-17]. In many of these studies, the higher-wavelength YAG laser appears to have an advantage over YSGG lasers, although the differences are often quite small [12-16]. These observations were supported by the present study; among the three laser systems, the Er:YAG laser with PIPS technique was superior to the Er,Cr:YSGG lasers in terms of tissue dissolution (*P* < 0.01). Although the difference in the wavelength of the Er:YAG and Er,Cr:YSGG lasers seems relatively small (2940 vs. 2780 nm), the effects on energy absorption and heat creation are considerable [12-16].

The differences in results between the lasers can also be attributed to the distinct mechanisms of action and tip design of the PIPS technique compared to other laser techniques. PIPS allows for lateral dispersion and propagation of the generated shock wave in NaOCl solution, leading to a photomechanical phenomenon that provides effective three-dimensional streaming of fluids [7]. These findings are inconsistent with a previous study that showed PIPS to be less efficient than Er: YAG laser with an endodontic fiber tip in tissue dissolution [7]. However, a direct comparison between the results obtained in the previous study and the present study was precluded due to differences in tested devices, methodology, and parameters measured. Nonetheless, both studies indicate that PIPS can enhance the tissue dissolution of NaOCl solution. Interestingly, under the same experimental conditions, Er,Cr:YSGG A laser in the present study performed slightly better than Er,Cr:YSGG B laser. There is currently no other published data available on the Er,Cr:YSGG A laser. 

In the field of endodontics, it is widely recognized that root canal irrigation is a crucial aspect of treatment, as it facilitates the removal of biofilms and necrotic tissue debris from the canal [2-4]. Various irrigation methods are employed, including traditional methods like syringe needle irrigation and ultrasound-facilitated irrigation, as well as newer, more energy-intensive methods such as lasers and multisonic irrigation [6-9]. However, there is a lack of information regarding the relative effectiveness of these methods in dissolving organic tissue in the canal, which is a key treatment goal. In this study, lasers and multisonic methods were compared for the first time, with conventional methods serving as an important reference point. The findings indicate significant differences in effectiveness among the various methods, with important implications for optimal clinical use. To serve as a proper control for the different active methods of irrigation, the study used a control consisting of the tissue target and a liquid medium, NaOCl, without any active irrigation or use of an energy source. This control allowed researchers to observe the effects of hypochlorite alone on the tissue, and serves as a standard example of the use of a control.

Almost all experiments on endodontic tissue dissolution published in the literature are done at room temperature [6-9,18,19]. This is due to the practical challenges of maintaining controlled temperatures in experimental settings. In clinical practice, the irrigant NaOCl is typically used at room temperature, although its temperature theoretically may increase during use in the root canal. However, with an irrigation flow rate of 5 mL/min, the irrigating solution in an average-sized canal is replaced in approximately 0.12 seconds [9]. While the exact temperature of the solution within the canal under these conditions is unknown, it is likely to be similar to or the same as the initial solution due to the short duration of exposure.

Currently, a consensus has yet to be reached regarding the selection of the optimal distance between the equipment tip and the tissue specimen in studies that compare the effectiveness of various irrigation methods in tissue dissolution. The variations in this study can be attributed to the guidelines provided by manufacturers for proper usage in the tooth/root canal setting. These recommendations aim to balance effectiveness and safety, particularly by minimizing the potential for the extrusion of caustic NaOCl into the periapical region. Therefore, the distance of the tip from the tissue sample accurately reflects the clinical scenario. According to current recommendations, the Er:YAG laser tips should be placed in the pulp chamber instead of the root canal [7], while for Er,Cr:YSGG lasers, the tip placement should be in the root canal [12]. Furthermore, a previous study has reported the formation of cavitation bubbles up to 4.5 mm distance from the tip for Er,Cr:YSGG lasers [20]. Therefore, it is reasonable to place the tips of Er,Cr:YSGG lasers 3 mm away from the tissue specimen to effectively exert their tissue dissolution effect. Accordingly, we chose a distance of 5 mm for the Er:YAG laser to differentiate the clinical application differences between the two methods. These manufacturer recommendations were reflected in the present study, with a distance of 5 mm for the Er:YAG laser and 3 mm for the Er,Cr:YSGG lasers. The handpiece of the GentleWave with CleanFlow technology sits on a sealed platform above the access opening and does not require a tip to be placed in the pulp chamber. Consequently, we chose a distance of 7 mm, which was longer than that of the Er:YAG laser (5 mm), to differentiate the clinical application differences between the two methods. In the case of open-ended needle irrigation, a jet is created towards the apex, extending even more than 3 mm apically to the needle tip [21]. Therefore, we chose to place the tip of the open-ended needle 3 mm away from the tissue specimen, consistent with the placement for Er,Cr:YSGG lasers. As for the ultrasonic tip, it was positioned 1 mm away from the tissue specimen. It has been reported that the acoustic streaming generated around the ultrasonic tip aids root canal debridement [22]. To avoid apical extrusion induced by ultrasonic activation, it is suggested to keep the ultrasonic tip at least 2 mm away from the apex [23]. This also highlights the extent of ultrasonic activation amplitude at a distance of approximately 2 mm. Therefore, placing the ultrasonic tip 1 mm away from the tissue specimen allows it to effectively exert its tissue dissolution effect. It is worth noting that placing the ultrasonic tip 3 mm away from the tissue specimen, similar to the lasers and the open-ended needle, does not provide a fair comparison among these irrigation methods.

A potential weakness of the methodology used in this study is the utilization of bovine muscle tissue instead of human pulp tissue for dissolution experiments. In this study, bovine muscle tissue was used to prepare tissue specimens with similar size, shape, and initial mass due to its fairly uniform composition. However, ideally, human pulp tissue should be utilized to test the tissue dissolution ability of different endodontic devices or irrigation solutions. Although there are histological differences between human pulp tissue and bovine muscle tissue, no studies have shown contradictory results between them when measuring dissolution by NaOCl [18,19]. Furthermore, standardizing the size and shape of tissue specimens is not feasible with human pulp tissue, especially when multiple irrigation techniques are tested with a large enough number of parallel experiments. Collecting enough human pulp tissue for numerous experiments in a short time is not feasible, which would further weaken the efforts to standardize the experimental conditions. Finally, obtaining bovine pulp tissue from slaughterhouses, which was widely used in the past, is no longer possible due to concerns about serious viral infections. The second weakness of this study is the use of a test tube model. While this model allowed for a visible, reproducible, and quantitative comparison of the target tissue, it differs in size and geometry from the root canal system, and fluid dynamics in the two environments may also differ to some extent. Despite attempts to create models with dimensions corresponding to the root canal system, it has not been possible to reliably stabilize the tissue in such models. Without tissue stabilization, suction in the MUS method interfered with the experiment, resulting in an unfair advantage for the MUS. Therefore, the results of this study should be extrapolated clinically with caution. Further studies are needed to compare the effectiveness of different irrigation techniques with NaOCl using different experimental designs.

## Conclusions

Despite its limitations, MUS with a CleanFlow handpiece and lasers was more effective at dissolving tissue than conventional irrigation. Additionally, Er:YAG laser with PIPS technology demonstrated superior tissue dissolution when compared to the Er,Cr:YSGG lasers.
